# Fertility in China: An uncertain future

**DOI:** 10.1080/00324728.2014.982898

**Published:** 2015-04-26

**Authors:** Stuart Basten, Quanbao Jiang

**Affiliations:** ^a^ University of Oxford; ^b^ Xi’an Jiaotong University

**Keywords:** China, family planning policy, fertility, one-child policy

## Abstract

As one of the world’s two population ‘billionaires’, the future of China’s population is truly of global significance. With its very low fertility and a rapidly ageing population, it might appear that the country’s famous (or notorious) family planning restrictions are somewhat anachronistic. Here, we explore the process of reform seen over the past three decades and, most recently, in late 2013. We suggest that the popular notion that the family planning restrictions are acting as a pressure valve suppressing a pent-up demand for childbearing, particularly in rural China, is likely to be inaccurate. We also suggest that further reform of the restrictions will not solve the problems of population ageing or many of the other issues widely associated with the restrictions. We conclude that the prospects for further reform are wide-ranging, but likely to be beset by many challenges.

In much of the academic and policy discourse about China, the country’s recent demographic history has been seen as inseparable from its population policies. From the ‘later, longer, fewer’ policy of the 1970s through to the more proscriptive regulations in place from 1979, China’s exceptional system of family planning restrictions has become the world’s best known—and most widely discussed—attempt to control population growth. The success of these attempts has had more than national significance. The country’s status as a population billionaire means that the future growth of its population, and therefore the impact of future policy intended to curb that growth, has important implications for the population of the planet. Plausible scenarios for global demographic change need to be informed by plausible assumptions about the effect of China’s future population policy.

## A rapidly ageing China

**Figure 1:**
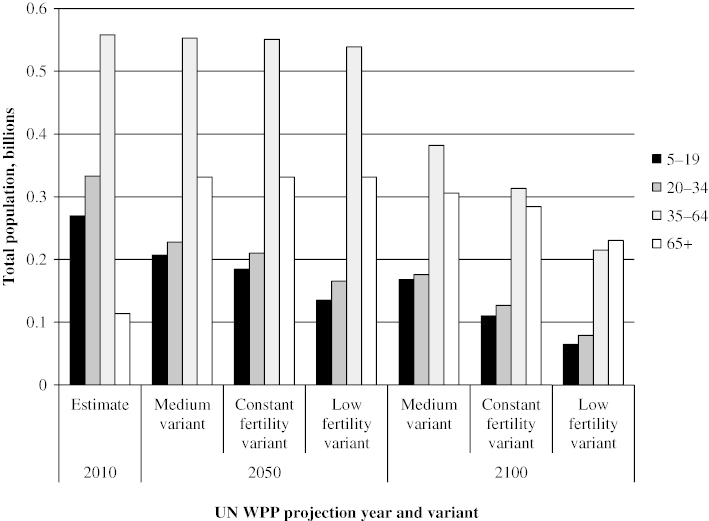
Projected total population size by key age groups, China *Note*: The constant variant implies a fixed TFR of 1.63 to 2100, while the low variant essentially entails a further marginal decrease and stabilization at around 1.3–1.4. *Source*: Based on the UN *World Population Prospects: The 2012 Revision* (UNPD 2013).

As a consequence of very low fertility rates and negligible international immigration, China is clearly ageing very rapidly (see, for example, Wang 2011; Cai 2012; Mai et al. 2013). The effect is evident in the changing relative size of the populations aged 20–34 and 65 and over, which can be seen in the United Nations (UN) population projections for 2050 and 2100 shown in Figure 1. The 20–34 age group is the crucial one in two ways: it is of childbearing age and the flexibility and recently acquired skills of its members are of critical value to the capabilities of the labour force. Currently this age group outnumbers the population aged 65 and over by about three to one. Under the UN’s medium fertility scenario (which assumes a consistent rise in total fertility rate (TFR) from 1.63 in 2010 to 1.88 by 2100) this relationship will reverse, so that by mid-century the ratio of the younger group to the older will be around two to three, and will continue to increase until the century’s end. However, the long-term prospects of prolonged low fertility—of the order seen in Japan, for example—would lead to a ratio of more than one to two within a couple of generations. Indeed, some believe that even these projections could be optimistic, in part because of doubts about the baseline assumptions used in making them. There is considerable current debate over the ‘true’ current fertility rate for China, not least between different government bodies (Gu and Cai 2011; Basten et al. 2014). In 2013, the National Health and Family Planning Commission (NHFPC 2013) officially stated that the national TFR was ‘1.5–1.6’, while others have suggested that the TFR is lower even than that, with Zhao and Chen (2011, p. 823) suggesting that TFR in the 2010 Census was ‘very likely to have been lower than 1.45’.

This transition will present a wide array of economic, social, and political demands to policymakers. For example, despite a shrinking labour force and further wage inflation, resources will have to be found to provide adequate care for the elderly and support for those of them in poverty (for details of these and other policy issues, see, for example, Heerink et al. 2011; Li et al. 2011; various chapters in Chen and Powell 2012; Du and Yang 2014). In so far as such problems are attributable to the very low fertility and consequent rapid ageing of the population, China’s anti-natalist policies may seem eccentric. Indeed, both Henry Kissinger and Joe Biden recently cited its family planning restrictions as a key factor in preventing China from sustaining the pace of its recent economic growth, with Biden referring to the ‘God-awful One-Child Policy’ (Rose 2011; Reuters 2012).

## So just ‘turn on the tap’?

Under these circumstances it is tempting to think that the current anti-natalist family planning policies can simply be relaxed further in order to send China onto a higher fertility trajectory. That would be expected to alleviate some of its future ageing problems (though it would almost certainly create many others, including pressure on the environment). The family planning restrictions, in this light, are simply an obstacle to fertility, which when removed will increase the supply of children. With this in mind, scholars have been tempted to produce forecasts based upon changes in fertility policy, on the assumption that families will have broadly the number of children they are *allowed* to have (Zeng 2007).

Over the course of 2012–13, a number of developments signalled that further relaxations in the family planning policy were in prospect. In 2012 the China Development Research Foundation—an influential think tank with close links to the National Government—recommended a ‘three-step’ policy transition: some provinces should implement a two-child policy straightaway, while a national two-child policy should be in force by 2015, and all limits on births removed by 2020 (Waldmeir 2013). In early 2013, China’s Population and Family Planning Commission was merged into the new Public Health and Family Planning Commission—a sign regarded by some as indicating a potential for further reform by shaking up entrenched bureaucracies (Jiang et al. 2013).

In November 2013 the Third Plenary Session of the Eleventh Central Committee of the Communist Party of China reiterated that family planning was a basic national policy, but changed the policy so that a couple in which one spouse was an only child would be eligible to have a second child (NHFPC 2013). This generated an intense period of worldwide media interest in China’s family planning policies. Following the announcement, provinces gradually implemented the policy, with almost all areas compliant by the summer of 2014 (Gu 2014). It has been estimated that around 15–20 million couples have now become eligible for a second child as a result of the reforms (Bai 2014). (Note that under the family planning regulations couples must *apply* for special dispensation to bear a second child if they are eligible to do so. Having the child without this licence is still, technically, a breach of the policy.)

The announcement was greeted with a strange mix of optimism and gloom as observers realized that while the ensuing baby boom would ameliorate the rapid pace of ageing it would also place an ever greater demand on public services. Share prices in baby formula and toy corporations spiked (but subsequently fell) (Steger 2013). Official estimates of 10 million extra births over the ensuing 5 years were widely quoted (Wetzstein 2014). The overwhelmingly common view was that there was a pent-up demand for children which only a careful process of gradual policy adjustment could manage. Zhai Zhenwu, a demographer from Renmin University, commented ‘A universal two-children policy will introduce a serious baby boom in a short period of time and put a lot of pressure on public services such as health and education’ (Zhuang 2014).

While it is too soon to know whether these hopes and fears will be justified by events, some indications of the number of extra births occurring since the inauguration of the policy is starting to emerge. In Xuanwei Prefecture (Yunnan Province), an area with a population of around 1.25 million people, there were just 36 applicants for a dispensation to bear a second child within the first 3 months of the reforms coming into effect. Local family planning officials blamed economic pressure on young couples for the low take-up (Liu et al. 2014). The projection by authorities in Nanjing City (Jiangsu Province) that around 9,000 such births would occur in 2014 has now been reduced to just half that number (Zhang and Yuan 2014). Finally, in Zhejiang Province, the first province to relax the regulations, the Health and Family Planning Commission had initially predicted that an extra 80,000 births would follow from the reform. By mid-July 2014, the Commission had received 43,100 applications, but only 2,444 extra births had followed. Wang Guojing, the Commission’s Deputy Director, has stated that they now expect just one-quarter of their initial estimate of extra births to occur (Zhuang 2014).

This evidence is only fragmentary, but it is consistent with what happened to birth rates after previous relaxations. The family planning policies have been reformed on a number of occasions. In 1984, for example, many provinces allowed couples in rural areas to have a second child if the first had been a girl. More recently, in many areas, if both husband and wife were only children they too have been allowed to apply for dispensation to have a second child. However, the take-up of this opportunity has generally been low. In Shanghai in 2012, for example, only around 8 per cent of couples eligible to have a second child by virtue of this rule actually did so (Wang 2012a). Hou and Ma (2008) and Hou et al. (2008) identified similar findings for Beijing. In each case, the number of couples who applied for dispensation to have the second child far exceeded those who actually went on to give birth.

To try to understand this phenomenon, a number of recent studies have examined fertility preferences in both urban and rural China (Whyte and Gu 1987; Choe and Tsuya 1991; Zheng et al. 2009). Some were studies of couples eligible to have a second child, while others used surveys to ask respondents to state their intentions under future scenarios of policy relaxation. According to recent reviews by Basten and Gu (2013) and Hou et al. (2014), preferred family size is declining and is negatively associated with education and income. As Hou et al. (2014) report, the mean desired number of children in 63 studies of urban fertility preferences in the period 2000–10 was just 1.50 (SD 0.25). The mean in 52 studies in rural areas over the same period was 1.82 (SD 0.36). While a number of caveats should be made about equivalence across studies in these meta-reviews, and about respondent bias (see Basten and Gu 2013, pp. 29–31), these findings appear to be robust. They are consonant with the results of other qualitative studies (e.g., Nie and Wyman 2005) and with data from nationally representative surveys.

In addition to these aggregate data, some studies have gone into greater depth to identify *why* fertility preferences are low. These show clearly that the core drivers are structural issues relating to the cost of living: the price of education, unreformed (domestic) gender roles leading to high opportunity costs of childbearing for females, and lack of adequate childcare facilities (Zheng et al. 2009; Merli and Morgan 2011). Whatever the effect of its unique history of population policies, China appears to be more like other countries in East and South-East Asia than had previously been thought in owing its low actual and preferred fertility to structural causes. All of this might mean that it is, indeed, a mistake to think that the family planning restrictions are a demographic ‘safety valve’ that is holding down an unmet need for children. By abandoning this safety valve metaphor, we can think more carefully about the complex interactions and processes which have underpinned the rapid fertility decline of the 1970s and that continue to the present day.

## Moving beyond an anti-natalist policy

Returning to the 2013 reforms, it appears clear that the policy adjustment was intended to increase the number of births in response to the rapidly ageing population. The switch from anti-natalist to pro-natalist policy (or, to be more precise, to a suite of family-friendly and work–life balance policies linked, explicitly or implicitly, to the issue of low fertility) is common to almost all low-fertility countries in Pacific Asia. In each case, however, a substantial period of time elapsed between reaching replacement-level fertility and making this switch towards pro-natalism. As Jones et al. (2009, p. 7) observe, anti-natalist policies were still in place in Taiwan, Singapore, South Korea, and Japan for up to 20 years after fertility fell below the replacement level. They attribute this slow turnaround to bureaucratic inertia, to a deficiency of theory regarding demographic transition (and the UN assumptions of fertility presented at the time), and to demographic momentum delivering continued population growth. Given that China has witnessed below-replacement-level fertility for 22 years now, could it actually switch over to a pro-natalist regime—or at least towards a more developed set of family policies which might encourage childbearing?

In truth, some local administrations have already made a switch towards a type of pro-natalism. In Shanghai, for example, the local Family Planning Commission began encouraging eligible couples to have a second child at least as early as 2009 (Branigan 2009). It was reported that ‘family planning officials and volunteers will make home visits and slip leaflets under doorways to encourage couples to have a second child if both grew up as only children … Emotional and financial counselling will also be provided, officials said’ (Wang 2012b). This observation from Shanghai clearly implies that the ideological and institutional shift from enforcing anti-natalist policies towards *encouraging* childbearing may not, after all, be so difficult. Furthermore, the sheer size of the family planning apparatus is such that a wholesale shift in ideology towards encouraging childbearing would yield an enormous number of local advocates and stakeholders.

Yet, as governments across East Asia (and elsewhere) are finding, raising fertility rates through policy is far from straightforward. The evidence suggests that the direct approach of the Shanghai family planning workers is unlikely to be successful (Jones et al. 2009; Frejka et al. 2010). As Peng (2007) observes, neither further relaxations of rules nor an ideological shift towards *encouraging* childbearing by the family planning apparatus are likely to succeed in achieving a sustained and significant increase in the number of births. Instead, it may be necessary to learn lessons from other low-fertility countries in Asia and beyond. The Chinese state and other stakeholders should tackle some of the real and perceived structural impediments that increase the direct and indirect or opportunity costs of childbearing. To do that it must promote ‘broad social change supportive of children and parenting’ (Jones et al. 2009, p. 8). Frejka et al. (2010) reach a similar conclusion in respect of other countries in Pacific Asia.

We should not be surprised, therefore, if some of the policies intended to improve the environment for childbearing (and, more directly, to increase fertility) seen elsewhere in East Asia are implemented—in one form or another—in China. These might include tackling the high cost of living and improving access to childcare, together with a number of other possible measures—more family-friendly work practices; reducing the high cost of housing for families; making cities more family-friendly; improving work–life balance; curtailing the excesses in educational expectations; altering views of gender roles; and so on. However, other more indirect measures to tackle the broader obstacles to childbearing could also be enacted. For example, under the current household registration system (*hukou*), families are often split up because parents work away from their homes and are unable to find adequate schooling for their children near their new residence. Improved services to families and children within the 200-million-strong ‘floating population’ of internal migrants could make family life easier for them. Increasing support for the elderly could lessen the burden on a generation of only-children couples. These couples, often finding themselves responsible for the care of two sets of parents and a child, are unsurprisingly reluctant to burden themselves with another baby (this is known in China as the ‘4-2-1 Problem’).

Yet any movement towards a more family-friendly social policy would certainly be fraught with difficulties. Firstly, demographic momentum has meant that even though the country has seen below-replacement fertility for over 20 years now (and negligible international immigration), total population has still grown by around 200 million and, crucially, is forecast to continue growing for another 20 years (UNPD 2013). It should also be remembered that China’s population has urbanized rapidly over this period, and over 70 per cent of its population is projected to be urbanized by the time population growth is expected to end in the 2030s (UNPD 2014). Because much of this population growth has been in densely populated areas, population growth has been highly visible in the development of massive cities and soaring property costs.

Secondly, there are significant vested interests that oppose change. One of the more important is the family planning apparatus, which has committed itself to anti-natalism for nearly two generations now. Performing the required ideological U-turn could be a challenge for some—not least because the collection of penalties for breaking the family planning restrictions has become a highly profitable enterprise for local administrations. Despite that, some local Family Planning Commissions are encouraging eligible couples to have a second child—a kind of ‘regional pro-natalism’ within national anti-natalist boundaries. This suggests that such an ideological shift is not impossible to achieve.

Finally, given that the bulk of family policy implemented in China since the ‘Open Door Policy’ in 1978 has focused on regulating ‘the rights and responsibilities of the family’ and ‘the welfare of children, women, the elderly, the vulnerable and the disabled’ (Xia et al. 2014, p. 257), it will be necessary to explore and learn new policies. Since the Chinese plan to recruit and train 1.45 million social workers by 2020 (Xinhua 2013), however, one should not underestimate the capacity of the Chinese state to implement ambitious social policies.

## Reform as a ‘silver bullet’?

So what of the future? It appears reasonable to assume that China is currently on track towards a ‘two-child policy’. We have suggested here that the *macro*-level impact of the 2013 reforms—and of those in the future—on TFRs and, ultimately, population ageing is likely to be relatively slight. Many studies have identified the reasons that people give for not having the total number of children to which they are eligible and for reporting fertility preferences of below two children.

This is not to say that further relaxation of the family planning restrictions will have no overall effect. Undoubtedly, any changes will be welcomed by many couples. While the number of extra births resulting from past relaxations may have been lower than expected, for many individual couples the opportunity to realize their fertility preferences legally will be a crucial development. Given the sheer size of the Chinese population, this is likely to lead to a modest ‘baby boom’ even if the relative proportion of eligible couples proceeding to have an additional birth will be low.

On the other hand, further relaxation will not be a remedy for many of the other problems that have been associated with the family planning policies. Studies by Short et al. (2001) and Goodkind (2011) on highly skewed sex ratios at birth emphasize the need to look beyond the family planning restrictions to consider other issues, such as differential reporting biases, differential stopping rules, long-standing son preferences, and so on (Basten 2012). The role played by the medical profession in providing illegal sex-selective abortion is often ignored and is surely an area requiring future policy attention. Only a profound national shift in attitudes towards females, the effective regulation of the medical profession, and movement towards a transparent, value-free demographic reporting system is likely to alleviate the problem of skewed sex ratios.

A similar policy challenge relates to the public image of the family planning restrictions. Egregious cases of coercion remain a problem, despite a ban on such activities in 1995 and again in 2002. There is also increasing evidence that local family planning officials are more interested in collecting fines, or the ‘Social Maintenance Fee’, to boost local revenues or to cover operating costs, than to persuade couples to adhere to their quota, or to cover the cost of extra public services that such ‘illegal’ children need (White 2006; Cao 2013). Recent estimates have suggested that such fees could total more than US$4 billion nationwide (Li 2012; Wei 2013).

These matters are of wider significance than the reformation of the family planning policy. The zeal of many officials for collecting fees for transgressing the policy is closely linked to the ways in which Chinese local government is financed. In 2001, the National Government reformed much of the agricultural tax system, resulting in a sharp decline in industrial–commercial taxes for local government. This, according to Tian (2008), changed motives for the collection of fines: in order to supplement the income of government, fines became less a tool to control births and more a device for local government to support their income following the reform of the agricultural tax system. Without reform—of the financial basis of Chinese local government, of the role of family planning administrations, and of the possibility of promotion through (over-)achievement of targets—such practices and abuses might continue. General Secretary Xi Jinping’s much-publicized crackdown on corruption in public office emphasized that the family planning policy is just one of many ways in which corrupt officials were able to abuse their position.

Finally, the selective enforcement of fines enables wealthy parents to evade the birth control policy. They can pay the fee comfortably or find loopholes—for example, by arranging for the mother to give birth in Hong Kong or Saipan (Basten and Verropoulou 2013; Li 2013). In 2013, Liu Daoping, vice-chairman of the National People’s Congress Standing Committee of Sichuan Province essentially admitted that the family planning restrictions were, in reality, only strictly imposed on the middle classes, because the destitute could not afford to pay and the rich could pay their fines easily (163.com 2013). This perhaps says more about the marked—and growing—inequalities in Chinese society than about the functioning of the family planning policies themselves.

## Conclusion

In January 2014, Zuo Xuejin, executive vice-president of the Shanghai Academy of Social Sciences, claimed that ‘by 2025, the government will be encouraging people to have more children’ (Kaiman 2014). In his city, we are already seeing family planning officials promoting childbearing among eligible couples. The recent reforms of the family planning policies have, undoubtedly, recognized that the *encouragement* of childbearing is, slowly but surely, entering the policy discourse in China.

Lieberthal and Oksenberg (1998, p. 3) describe different models of policy formation in China. In one model a policy is adopted with the intention of solving new policy problems pressing upon leaders. Examples might include the original family planning policies implemented in the 1970s and 1980s. In another model a ‘policy could be promulgated in order to keep alive the ideological vision of its proponents’. Here a clear parallel can be drawn with the ongoing reforms of the family planning policy over the past three decades and the explicit statement in the 2013 reforms that the family planning policy was still a ‘basic national policy’. The recent further relaxation of the family planning restrictions in response to new ‘pressing’ policy problems of a rapidly ageing society suggests that the formation of China’s population policy is shifting from the second to the first model described by Lieberthal and Oksenberg. If this is the case, the once unlikely prospect of a Chinese *pro*-natalist policy could not be too far off. However, the importance of maintaining the ‘ideological vision’ should not be ignored.

Furthermore, as Lieberthal and Oksenberg (1998) themselves observe, this simplified view of the Chinese policymaking process does little justice to the complex web of a multiplicity of stakeholders involved and the structure of Chinese bureaucracy and decision-making. As well as policy promulgations which reward ‘networks of loyalists’ or which are ‘tactical ploys’ to rebuff challenges from rivals, Lieberthal and Oksenberg identify a bargaining model of policy formation. Here, ‘Policy *X*’ results from ‘a bargain between Ministries *A*, *B*, and *C* and Province *D*’ which is ‘(a) brokered by one or more top leaders’, ‘(b) arranged by co-ordinating staffs acting in the name of one or more top leaders’, or ‘(c) negotiated by the supra-ministry co-ordinating agency, and ratified through routine procedures by the top leaders’ (pp. 8–9). Given the important stakes held by assorted ministries and provinces in the future size and characteristics of the Chinese population and the presence of a new reformed Public Health and Family Planning Commission, we might expect to see future reforms emerging from this bargaining between stakeholders.

It is worth highlighting two final notes of caution on this possible trajectory of policy relating to family planning. The first refers to the policymaking process in China. Returning to Lieberthal and Oksenberg’s bargaining model of policy formation, they note that ‘disgruntled Ministers *D* and *E* [or other stakeholders]’ as losers in the deal may begin to pursue ‘strategies to erode the agreement’. In response to this, further bargains are sought to ‘reconcile the conflicting organizational missions, ethos, structure, and resource allocation of the ministries involved’. Thus, as they conclude, ‘policies are not necessarily either coherent and integrated responses to perceived problems or part of a logical strategy of a leader or faction to advance power and principle’. The nature of Chinese policymaking is such that the development of any kind of holistic population policy is likely to pose challenging problems for all involved.

The second note of caution concerns the apparent normalization of small family sizes in China. Have the family planning policies, based on the ‘sanctity’ of the principle of population control and the limitation of childbearing, created new, enduring norms? If so they would have a very long-term impact (Wang et al. 2013), and the complexities of China’s local and national politics could have a substantial effect on world population.

Judging by the experience of some of its neighbours, and with these two caveats in mind, the Chinese state may well find that it is much easier to ‘encourage’ people to have fewer children than to have more.
